# Obstacle Classification and 3D Measurement in Unstructured Environments Based on ToF Cameras

**DOI:** 10.3390/s140610753

**Published:** 2014-02-14

**Authors:** Hongshan Yu, Jiang Zhu, Yaonan Wang, Wenyan Jia, Mingui Sun, Yandong Tang

**Affiliations:** 1 College of Electrical and Information Engineering, Hunan University, Changsha 410082, China; E-Mails: jiang126@126.com (J.Z.); wang_yaonan@hotmail.com (Y.W.); 2 Laboratory for Computational Neuroscience, University of Pittsburgh, Pittsburgh, PA 15213, USA; E-Mails: jiawenyan@gmail.com (W.J.); drsun@pitt.edu (M.S.); 3 State Key Laboratory of Robotics, Shenyang 110016, China; E-Mail: ytang@sia.cn; 4 College of Information Engineering, Xiangtan University, Xiangtan 411105, China

**Keywords:** mobile robotic navigation, obstacle detection and classification, time-of-flight camera, region of interest detection, unstructured environment perception

## Abstract

Inspired by the human 3D visual perception system, we present an obstacle detection and classification method based on the use of Time-of-Flight (ToF) cameras for robotic navigation in unstructured environments. The ToF camera provides 3D sensing by capturing an image along with per-pixel 3D space information. Based on this valuable feature and human knowledge of navigation, the proposed method first removes irrelevant regions which do not affect robot's movement from the scene. In the second step, regions of interest are detected and clustered as possible obstacles using both 3D information and intensity image obtained by the ToF camera. Consequently, a multiple relevance vector machine (RVM) classifier is designed to classify obstacles into four possible classes based on the terrain traversability and geometrical features of the obstacles. Finally, experimental results in various unstructured environments are presented to verify the robustness and performance of the proposed approach. We have found that, compared with the existing obstacle recognition methods, the new approach is more accurate and efficient.

## 1. Introduction

Nowadays, mobile robots, such as the unmanned ground vehicle (UGV), rescue robots, and space robots, are being increasingly utilized in complex and unstructured environments extending from the traditional indoor or man-made environments. With the increasing environmental complexity, fast and robust 3D environment perception and recognition have become extremely important in autonomous robotic navigation.

**Figure 1.**
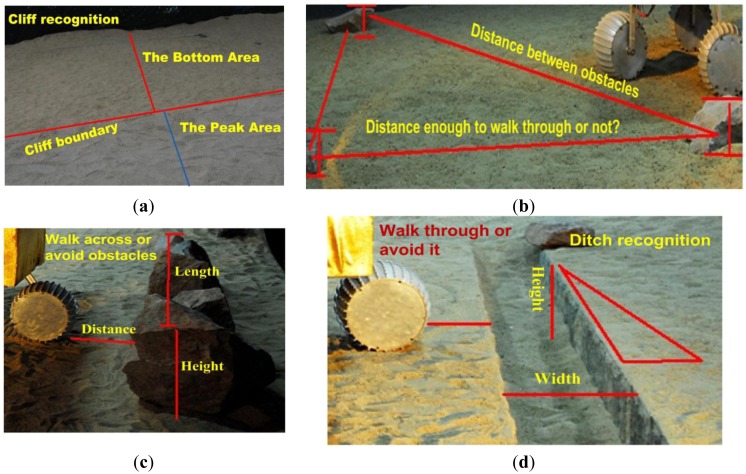
Some specific navigational tasks for robots in unstructured environments. (**a**) Cliff recognition; (**b**) Multiple obstacle detection and geometrical relationship measurement; (**c**) Stone recognition and 3D information measurement; (**d**) Ditch recognition and 3D measurement.

There are three key requirements for a field robot to make an optimal trade-off between efficient navigation and safety: (1) as illustrated in Figure 1, a robot should detect obstacles in its heading direction in real-time. Compared with a structured environment, unstructured environments are usually more complex and have less static and obviously distinguishable features, such as planar surfaces, country roads and other easily recognizable field features. A more realistic case is shown in Figure 1a, where there are two regions with large height differences. However, there are no obvious image features, such as color, texture, intensity *etc*., to distinguish the height differences from each other. In addition, there are more noises and environmental disturbance, such as dramatic light changes. Consequently, conventional vision based navigation methods have significant limitations in these complex environments; (2) compared with the indoor robot, a field robot usually manipulates several types of motion primitives, each of which is adapted to a specific terrain type. As illustrated in Figure 1, there are different types of obstacles, such as stones, rocks, ditches, cliffs, *etc*. Field robots must have obstacle recognition ability to help the planner select the most appropriate navigational behavior; (3) a field robot usually has a certain cross-country ability, such as walking over stones, rocks, and ditches as well as climbing. It is necessary to acquire the 3D information of the obstacle for autonomous navigation. For the case where obstacles are present in the heading direction, such as the stones or ditches illustrated in Figure 1b,c, the robot needs information about heights and widths of obstacles to decide whether to avoid or walk over them according to its mechanical capability. For the situations where a robot faces multiple obstacles as illustrated in Figure 1d, the robot needs to calculate relative distances between neighboring obstacles to determine whether the obstacles can be overcome and which travel direction is optimal.

The existing stereo-vision or range-sensors can hardly provide the complete information about 3D environments in real-time, so field robots still have great difficulties in autonomous navigation. The Time of Flight (TOF) camera is a 3D visual sensing system that captures image along with per-pixel 3D space information. Therefore, the TOF camera has great advantages in assisting terrain perception and recognition. This paper proposes an obstacle detection and classification method based on the use of TOF Camera-SR3000 manufactured by Mesa Imaging AG (Zürich, Switzerland). By modeling human walking mechanisms, obstacles are firstly segmented from the scene as regions of interest using both the intensity image and the 3D data provided by the SR3000 camera. Based on the 3D geometry parameters of obstacles, a multi-RVM classifier is designed to classify obstacles into four classes consisting of ditches, rocks, slopes and stones. The novelty of this work lies in fast and robust obstacle recognition in unstructured environments without prior knowledge, while providing valid and dense 3D measurement of obstacles.

This paper is organized as follows: in Section 2, relevant literatures are reviewed. Section 3 describes the proposed approach in detail. Section 4 presents experimental results and data analysis. Section 5 is a comparison and discussion about the proposed method. Finally, conclusions are given in Section 6.

## 2. Related Works

### 2.1. 3D Sensors for Robot Navigation

Currently three kinds of sensor technologies are mainly used in 3D sensing of mobile robots, including laser sensors, stereo cameras and 3D range cameras.

#### 2.1.1. Laser Sensors

Sensors using lasers to acquire 3D data can be classified into actuated laser ranger finders and 3D laser ranger finders [1]. Typically, an actuated ranger finder is a 2D sensor attached to a platform rotated by a servomotor [2–5]. Some groups use two 2D laser rangefinders to enhance performance [6,7]. In order to receive consistent 3D data with such sensors, a stop-and-go mode for traveling is necessary, and multiple consequent scans are taken and merged. The precision of 3D data points depends on the resolution of servomotor and on the precision of the scanner. The main disadvantage of actuated 2D laser ranger finders is that they are slow and the scan is time-consuming.

The 3D laser range finders can provide high-resolution data at a very high sampling rate. Examples of this type of range finders include RIEGL, CYRAX, zoller + fröhlich scanner, Velodyne 3D-LRF, and space ground LiDAR, which all generate consistent 3D data points within a single 3D scan. The major benefits of these sensors are high resolution and large field of view. Unfortunately, these sensors are usually heavy in weight, with high power consumption and high cost. In contrast to expensive 3D laser sensors, the FX 3D LIDAR sensor is relatively lightweight and has no rotational mechanical parts, but its resolution is low.

#### 2.1.2. Stereo Vision Systems

Stereo vision systems acquire 3D data with two or more cameras by passive sensing. Some commercial stereo vision systems are available, such as those manufactured by PointGray and VidereDesign. Comparing to laser ranger sensors, the range data resolution of a stereo camera is usually lower. However, it can provide high-resolution images, texture and color information, not just a 3D geometry. Now, stereo cameras have been widely applied to map-building [8–12], natural environment navigation, and rescue missions [13]. Nevertheless, stereo vision has limitations, especially in environments with few features and limited visibility due to undesirable light conditions. Furthermore, stereo vision is effective only in the near field as its range precision and maximum depth is limited because its range resolution usually decreases rapidly as the depth increases.

#### 2.1.3. 3D Range Camera

3D range cameras have been developed for 3D data gathering providing both an intensity image and a range image. A TOF camera has an active sensor that determines both the range and intensity information at each pixel by measuring the time taken by infrared light beam traveling to the object and reflected back to the camera [14,15]. Now several manufactures have begun marketing of these cameras, such as MESA Imaging, PMD Technologies, and 3DV Systems. Microsoft Kinect is another kind of popular range camera based on the light coding technology. Its ability to fast and reliable access to 3D data at high frame rates has enormous potential applications in mobile robotic mapping [16–20], navigation [21,22] and localization [23], industrial robot collision avoidance [24,25], human tracking [26], special measurement [27].

In general, the distance accuracy and image quality of TOF cameras is still relatively low compared to that of other 3D sensors. External as well as internal factors which affect the camera performance are integration time, target reflectivity, object distance, movement artifacts, environmental conditions (temperature, light, multiple reflections, non-ambiguity range) and so on [28–30]. Signal noise reduction is usually conducted by either averaging information over time or by using spatial smoothing filters [30,31]. Some calibration methods have been presented for range error rectification [31–33] caused by object reflectivity, distance and *etc*. Low integration time is often used for reducing the blurring effect caused by motion, but it is necessary to compensate for the low SNR of a single image. One possible approach is to fuse data from multiple frames recorded at a higher frame rate to improve SNR of the averaged signal [28]. Lindner *et al.* introduced a solution for motion artifacts of ToF cameras based on optic flow techniques [34].

Meanwhile, researchers have proposed lots of methods to enhance the resolution in either the 3D depth image or the 2D image. Some of the methods are based on the combination of higher resolution image and depth image acquired by a 2D camera and a ToF camera [35–37], while other methods are based on the fusion of a stereo camera and a ToF camera [38,39]. Yuan *et al.* [40] proposed a laser-based navigation system enhanced with 3d time-of-flight data. With the development of 3D range cameras, this has a great future in an enormous range of applications.

### 2.2. Obstacle Detection Based on 3D Information

Compared with measurement data from 2D laser sensors or sonar sensors, 3D data acquired by 3D sensors contains a large amount of information about the environment which may correspond to obstacles, drivable surfaces (ground), or objects that the robot cannot reach [41]. Consequently, the extraction of safe navigation areas is done by calculating the probability of each mesh point belonging to the ground surface (safe navigation zones) or to an obstacle [5,21,40]. The plane-based obstacle detection approach states that the environment's ground can be modeled by planes and obstacles are considered to be these 3D points standing out of the estimated ground planes [42–45].

Using 3D range data to fit a least squares plane as the ground plane is the most direct and simple method [46,47]. Other fast and robust methods determine the planarity of the area by the surface normal and the offset distance from the sensor origin [1,4]. This plane-based approach can also be applied indirectly, such as through an estimated homography [48] or through a Hough space of planes [49]. The plane-based approaches for obstacle detection are highly appealing because of their high computational efficiency.

Unfortunately, most terrain environments are not perfectly planar, small variations in height on uneven terrains are often confused with small obstacles. In those situations obstacles are better defined in terms of geometrical relationships between their composing 3D points [50,51]. Gor *et al.* [46] used intensity information to detect small rocks and height distance between object to the ground plane to detect large rocks in the image. Santana *et al.* [52] presented a stereo-based all-terrain obstacle detection method using visual saliency. Another obstacle detecting approach is to use heuristics in the form of local point statistics obtained directly from a 3D point cloud [53].

### 2.3. Obstacle Classification Based on 3D Information

There are two major approaches to obstacle classification, including semantic classification and geometric parameter-based classification. Lalonde [3] presented a semantic classification method using visual saliency features to classify 3D point clouds of terrain containing vegetation into three classes: “scatter”, “linear” and “surface”. Procopio [54] introduced a near-to-far learning method for terrain classification with a stereo camera. For geometry parameters based obstacle classification, Lux and Shaefer [55] yields map of obstacles based on range information of 3D laser sensors, in which a horizontal section of the scene is labeled as traversable, inaccessible and undetermined, respectively. In order to classify the scene with further meaning, 3D maps are often introduced to acquire more geometry parameters [56]. Further possible 2.5D representations are elevation maps, such as digital elevation models (DEM) and digital terrain models (DTM) [10,57]. Saitoh *et al.* presented an Inclined Surface Grid (ISG) map representation which stores height, slope (roll and pitch angle) and roughness information [58]. Some literatures [6,59] introduce a goodness map, and each cell of the map contains the height, roughness, step heights and traversability information. Rusu *et al.* [60] presented a hybrid 3D map representation from octree cells and polygonal modeling with semantic labeling for ground, vertical, level stairs and unknown areas. Compared to the above methods, a 3D polygonal representation of a planar surface directly extracted from 3D point clouds is more efficient in computation and memory storage, while it can represent structured object [4,19,54]. However, it is limited to natural environments with unstructured objects, such as trees, round slopes, *etc*.

## 3. Proposed Method for Obstacle Detection and Classification

### 3.1. General Scheme

Visual attention, which rapidly detects certain parts of interest in a given scene, plays a fundamental role in human vision [61]. 3D vision is an intrinsic component of biological vision, and depth information obtained is critical in detecting objects of interest [61,62]. Generally, in order to avoid collision, humans need pay attention to nearby regions with obstacles at a relevant distance and in meaningful dimensions, particularly those at the ground level, while navigating in a complex environment. If there is an obstacle, an obstacle-free ground path is selected for navigation without collision. The non-relevant regions, such as those above the person's head (e.g., sky), distant objects and a flat ground, do not affect a selected path of movement. For humans, those regions are extracted based on the spatial information obtained from both eyes. Since the ToF camera provides 3D information like that in the human vision, our navigational system utilizes 3D spatial information and classifies the scene into both irrelevant regions and regions of interest (ROI) as defined in Table 1, inspired by observing the characteristics of the human visual system.

**Figure 2.**
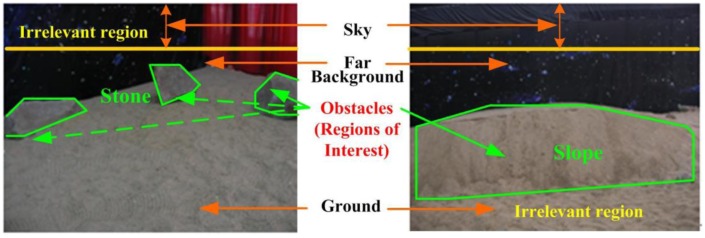
Region of interest and irrelevant region in unstructured environment.

According to this definition, sky, ground and far background do not affect the route of a mobile robot. Therefore, we call the corresponding regions irrelevant regions. The robot focuses on detecting obstacles as regions of interest (ROI), such as stones, slopes, *etc*. rather than irrelevant regions as illustrated in Table 1 and Figure 2.

**Figure 3.**
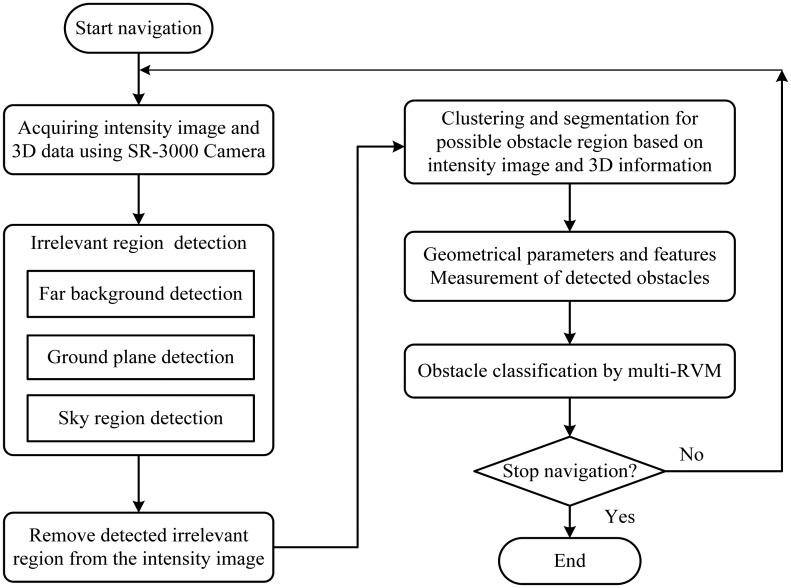
General scheme of the proposed method.

As obstacles are often irregular in shape and have undefinable features, it is difficult to detect and recognize them directly, especially in the unstructured environment. However, we can extract irrelevant regions of a scene according to the definition in Table 1 (see detail in Section 3.2.2) after the 3D information of this environment becomes available. We thus propose a different obstacle detection method by removing irrelevant regions from the environment using the 3D information of the SR3000. The proposed method performs obstacle detection and recognition in a procedure illustrated in Figure 3. It can be divided into three key parts, 3D information acquiring, obstacles detecting based on ROI and obstacle recognition-based RVM, which will be presented in detail in the following sections.

### 3.2. Obstacle Detection Based on TOF Camera

A self-developed robot equipped SR3000 camera is used in this paper. A SR3000 camera captures intensity images in the 176 × 144 resolution along with the per-pixel 3D space information. To describe the proposed method clearly, some notations are defined as follows. The intensity image from the SR3000 camera is defined as *GI*, and the corresponding 3D data is [*X*, *Y*, *Z*]. For a space point P with projection pixel coordination (*i*,*j*) on a SR-3000's intensity image, *GI*(*i*,*j*) stands for the intensity value; Referred to the SR3000 camera coordinate system, *X*(*i*,*j*) is the value in horizontal x-axis direction of space point P, *Y*(*i*,*j*) is the height value in the vertical y-axis direction, *Z*(*i*,*j*) is the distance value in z-axis direction.

#### 3.2.1. 3D Information Acquiring and Preprocessing

**Figure 4.**
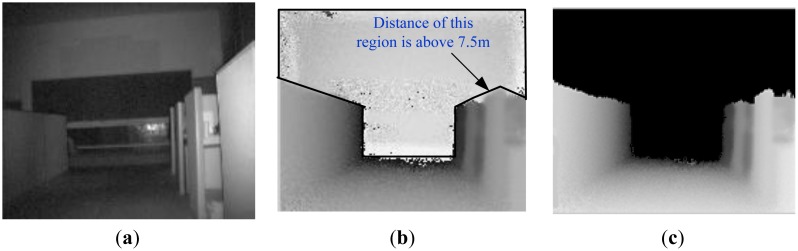
“Back folding” measurement filtering in lab scene based on proposed method [65]. (**a**) Gray image of lab scene (**b**) Depth image of lab scene (**c**) Depth image after processing.

**Figure 5.**
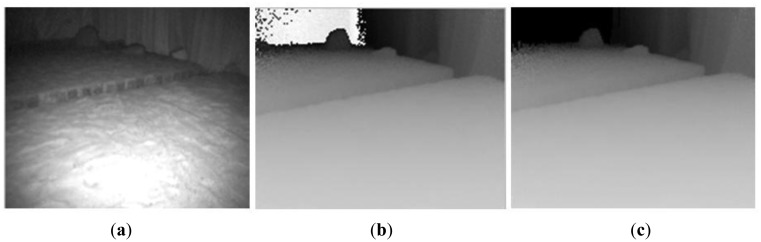
“Back folding” measurement filtering in ditch scene based on proposed method [65]. (**a**) Gray image of ditch scene c (**b**) Depth image of ditch scene (**c**) Depth image after processing.

(1) Camera parameters setting and 3D Information acquiring
  Since the integration time of ToF cameras has a direct effect on signal-to-noise (SNR) of measurement, accuracy of measurement, movement artifacts, and so on, it should be selected based on the required acquisition speed, the measurement quality and measured objects. In our case, we select integration time based on the following facts:
  First, as we focus on obstacle detection and classification, the environment is simplified in our study. Only static obstacles in the scene are processed. Furthermore, the robot operates in stop-go mode when capturing a scene by ToF camera. Therefore, movement artifact in our measurement can be ignored.
  Since ToF camera measurement is based on the received modulation signal, the accuracy of distance measurement of a ToF camera depends heavily on the signal-to-noise of the received signal. Longer integration time allows one to capture a larger amount of reflected light, which results in lower noise. In addition, the requirements for integration time are different for different objects. To reach the same measurement quality, a longer integration time needs to be set for low-reflective and further objects than for high reflective and closer objects. Consequently, long integration time is necessary to ensure the accuracy of measurement with less noise. However, longer integration time also induce higher temperature of the sensor. Furthermore, very long integration time leads to saturation.
  Based on the above mentioned facts and the existing analysis [28–32], different integration times were tested and compared in our test environments, and 80 ms was finally selected for SR3000 in our case to get measurement data with constant accuracy and less noise.
  Furthermore, to reduce the amount of heat generated, the camera is operated in triggered mode rather than continuous mode. The focus of SR3000 camera is fixed during the whole navigation process. In our situations, as shown in Section 4, interesting obstacles are usually in the range of 2 m–5 m. As a result, we set the focus in the distance of 3.5 m. Additionally, in order to improve the distance accuracy, we calibrate the measurement data according to the neural network based approach described in reference [33].
(2) Noise reduction based on spatial smoothing
  Besides the integration time, noise in images obtained by the SR3000 camera is reduced using a spatial smoothing filter. Compared with median filter and Gaussian filter, the bilateral filter [63,64] takes into account the difference in value within the neighbors to preserve edges while smoothing. Therefore, it can filter out random noises of distance information inside the object region while keeping edges of the object. Consequently, the bilateral filter is introduced for distance measurement denoising before obstacle detection and measurement.
(3) “Back folding” measurement filtering
  Measurements by the SR3000 are also subject to a so-called “ambiguity” or a “back-folding” phenomenon. When the distance of object is further than the ambiguity range (here 7.5 m), the measurement is usually a random value in range of 0∼4 m with a low amplitude instead of a true value. The possible solution is to filter those values out by setting an amplitude threshold as objects situated outside the non-ambiguity range. However the amplitude threshold is difficult to define due to the difference of scene and object characteristics.
  In the robot developed by us the ToF camera is mounted horizontally. Consequently, along the vertical direction in ToF images, pixels with a longer distance usually appear in the upper area compared with pixels with a shorter distance in an open or obstacle free environment. If pixels do not hold this feature, they should belong to a valid obstacle or a “back-folding” region. In addition, valid object region should satisfy the principle of image spatial continuity and deep information continuity, and its range or image information should be continuous instead of random. Based on this knowledge and the characteristics of amplitude, we have presented a method to remove those noisy measurement caused by “back folding” phenomenon [65]. As shown in Figures 4 and 5, regions with ambiguity measurements are filtered out using our proposed method, which effectiveness has been verified.

#### 3.2.2. Irrelevant Region Detection

**Figure 6.**
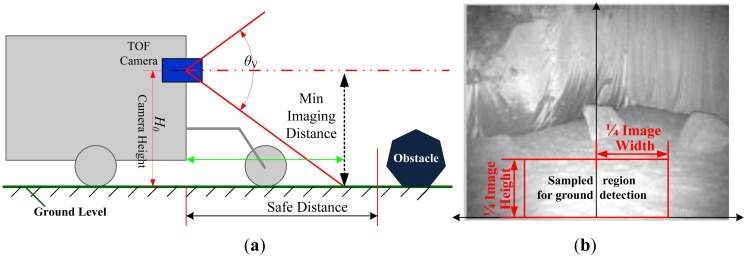
The principle of safe region for robot. (**a**) Robot's configuration and safe region definition. (**b**) The sampled region for ground detection.

(1) Far background detection
  According to the above definition, a pixel (*i*,*j*) on intensity image GI falls within a far-background region if its distance value *Z*(*i*,*j*) is more than a distance threshold *D_FB_*. As a result, the point is removed from the intensity image by setting its intensity value to zero:
  (1) $$\begin{array}{cccc} \text{If} & Z(i,j)>D_{FB} & \text{then} & GI(i,j)=0 \end{array}$$
(2) Ground plane detection
  In order to calculate the accurate ground plane, a simple and fast histogram based method is presented. The TOF camera is mounted horizontally on the robot. As a result, the ground plane means the points on the plane have a constant height.
  As illustrated in Figure 6a, generally the area adjacent to the robot in the forwarding direction is the safest area for safe navigation, and the lower center region in the intensity image will also likely be a safe region for the robot. Consequently, as shown Figure 6b, we select a sample region (SR) in the intensity image to calculate the parameters of ground plane because ground plane will appear in this area with the highest probability.
  For a given pixel (*i*,*j*) on the intensity image of the SR-3000 camera, we can draw its corresponding 3D information provided by *P*(*x*,*y*,*z*). Therefore, we express the height of any point in the SR region as *Y*(*m*,n). However, the ground is not perfectly planar in most terrain environments. Small variations in height on uneven terrains are commonly encountered. Consequently, we assume that the height of points on the ground plane has a Gaussian distribution $N(H_{g},\sigma_{H}^{2})$, where *H_g_* is the average ground plane height, and σ is the standard deviation of the ground height. The parameters of ground plane are calculated as follows:
  (a) As the camera is mounted horizontally on the robot with height *H*_0_ (the distance between the center of camera to the ground), we defined the heights of points in the SR region in the range of [*H*_0_ −10, *H*_0_+10] (in cm), for all possible points in a ground plane set, denoted by PG.
  (b) For a point in PG, we calculate its height distribution histogram. The horizontal axis X in this histogram stands for the height with step of 2 cm, and the vertical axis Y stands for the number of points drops in the height range [*H*(*i*),*H*(*i*+1)] represented as *PNum*(*H*(*i*)).
  (c) We select the top three scores in the histogram as *PNum*(*H*(*i*)), *PNum*(*H*(*j*)) and *PNum*(*H*(*k*)), calculate the sum of points in one neighborhood and select the maximal one. Then, the more accurate height range of ground plane is defined as follows:
    (2) $$\begin{array}{ll} if & \overset{1}{\underset{w=-1}{\text{∑}}}\text{Pnum}\left(H(j+w)\right)=\text{Max}\left(\overset{1}{\underset{m=-1}{\text{∑}}}\text{Pnum}\left(H(i+w)\right),\overset{1}{\underset{m=-1}{\text{∑}}}\text{Pnum}\left(H(j+w)\right),\overset{1}{\underset{m=-1}{\text{∑}}}\text{Pnum}\left(H(k+w)\right)\right)\\ \text{then} & \text{GRange}=[H_{0}-10+2*(j-1),H_{0}-10+2*(j+1)] \end{array}$$
  (d) The parameters of ground plane are calculated as follows:
    (3) $$H_{g}=\frac{\overset{M}{\underset{i=1}{\text{∑}}}\overset{N}{\underset{j=1}{\text{∑}}}Y(m,n)}{K},\forall Y(m,n)\in \text{GRange}$$
    (4) $$\sigma =\frac{\overset{M}{\underset{m=1}{\text{∑}}}\overset{N}{\underset{n=1}{\text{∑}}}{\left(Y(m,n)-H_{g}\right)}^{2}}{K},\forall Y(m,n)\in \text{GRange}$$
    where K is the number of points with height in the range of *GRange*.
  (e) As the parameters of ground plane are calculated, the points on the ground plane are defined. Pixel (*i*,*j*) of intensity image GI with height value *H*(*i*,*j*) falls in the range of [*H_g_*−2*σ*,*H_g_*+2*σ*] will be considered as a ground plane point. In order to remove the ground points from the intensity image for further obstacle detection, the corresponding intensity value of ground plane point is set to 0, as illustrated in Equation (5):
    (5) $$\begin{array}{cccccc} If & Y(i,j)\in [H_{g}-2\sigma ,H_{g}+2\sigma ] & \text{then} & (i,j)\in \text{GPange}e & \text{and} & GI(i,j)=0 \end{array}$$
  For different complex environments, the ground plane can be detected with high robustness and accuracy, and most points on the ground plane are removed from the scene. However, there are still some undetected ground points with considerable height variance in the image, which need to be removed by further processing (to be described in Section 3.2.3).
(3) Sky detection
  The sky region is defined as the region where which height is far beyond what the robot can reach. As the ground plane is detected, we define the sky region according to the height difference with ground plane height. A pixel (*i*,*j*) in the intensity image GI with height *Y* (*i*,*j*) elongs to the sky region if its height is higher than the ground plane with threshold *δ_S_*, and then the corresponding intensity value of the pixel is set to zero:
  (6) $$\begin{array}{cccc} \text{If} & Y(i,j)-H_{g}>\delta_{S} & \text{then} & GI(i,j)=0 \end{array}$$

#### 3.2.3. Obstacle Detection Based on Intensity Image and 3D Information

(1) Segmentation of regions of interest
  According to the definition in Table 1, the remaining pixels in the intensity image GI after removing irrelevant regions are regions of interest. This fact immediately suggests the use of binary images to represent these regions. A binary image has an intensity value 0 for the irrelevant regions and 1 otherwise:
  (7) $$\begin{array}{cccc} \text{If} & GI(i\cdot j)>0 & \text{then} & GI(i\cdot j)=1 \end{array}$$
  In practice, the detected ground plane often contains unwanted “noisy parts” because of the coarseness and surface irregularity of the ground. A morphological filtering method is introduced to suppress noises. As given in Equations (8) and (9), erosion followed by dilation with the same structuring element B is used to remove isolated small objects or noises from image GI:
  (8) GI∘B=(GIΘB)⊕B
  (9) GI•B=(GI⊕B)ΘB
(2) Region of interest clustering and obstacle detection
  After extracting obstacles, some parts of the obstacles are often overlapped with each other in the intensity image. Naturally, obstacles should be individual objects (such as rocks, slope, and so on). For any individual obstacle, we know that adjacent points in the intensity image must also be adjacent in spatial relation. Consequently, detected interested points can be clustered into separate obstacles on the basis of their geometric information (*i*.*e*., depth and horizontal information) and connectivity.
  Comparing adjacent points located in the obstacle region, if the distance between two points stays within a certain range, we assign them to the same category. Otherwise, we assign them to different categories. Then, a current point is regarded as starting point of new category and compared with the rest points. In this paper, the Manhattan Distance is selected to calculate distance between two points as follows:
  (10) $$D_{i,j}=c_{W}\left|X(i_{1},j_{1})-X(i_{2},j_{2})\right|+c_{H}\left|Y(i_{1},j_{1})-Y(i_{2},j_{2})\right|+c_{D}\left|Z(i_{1},j_{1})-Z(i_{2},j_{2})\right|$$
  Where *c_W_*, *c_H_* and *c_D_* represent contributions to clustering, which is determined by spatial changes in horizontal, vertical and depth directions. For the obstacles, change of distance in the depth direction is usually more obvious, thus we have *c_W_* < *c_H_* < *c_D_*.

### 3.3. Obstacle Recognition with Multi-RVM

Geometrical information is the basis of obstacle classification. However, irregular shapes of obstacles and their multiple geometrical features do not allow clear distinction rules to be defined. As a result, definition of each class is overlapped and ambiguous in some extent. Consequently, it is difficult to build an exactly mathematical classifier to differentiate obstacles into multiple classes. It has been shown that the support vector machine (SVM) and relevance vector machine (RVM) methods are effective in tackling those classification problems. We thus adopt a multi-RVM method to perform accurate and robust obstacle recognition.

#### 3.3.1. Obstacles to be Recognized

In Section 3.2, obstacles are clustered and detected. To realize navigation in a natural environment, it is important to recognize obstacles and plan different efficient navigation strategies accordingly. It is assumed that the robot can climb step-like obstacles with a height threshold H_rc_ and slope obstacles with a slope angle threshold *θ_rc_*. However, the robot can walk across obstacles higher than H_rc_ if the obstacle region has a certain slope angle. In this paper obstacles are expected to be fall into four categories for navigation purposes, based on the movement performance of the robot.

(1) Rock: Small obstacles (rocks or steps) can be traversed by robot without avoiding them according to movement performance of robot. In addition to help distinguishing them from other obstacles, the maximum height of a rock obstacle is defined as H_r_ which is usually higher than H_rc_.
(2) Stone: Compared with rocks medium obstacles such as stones, pillars and trees must be avoided by robots. The *Width* and *D_depth_* of stone obstacles should be smaller than the width threshold of slope (W_sl_) while its height is above the threshold H_rc_.
(3) Slope: huge obstacles, such as a slope, wall or a megalith (certainly, whether a slop can be climbed is determined by its slope angle and mechanical properties of the robot). Its height is higher than H_r_ while its minimum width is defined as W_sl_ which is usually wider than the robot's width.
(4) Ditch: obstacles below the ground plane, such as a ditch or a small water path.

#### 3.3.2. Features Used for Obstacle Recognition

**Figure 7.**
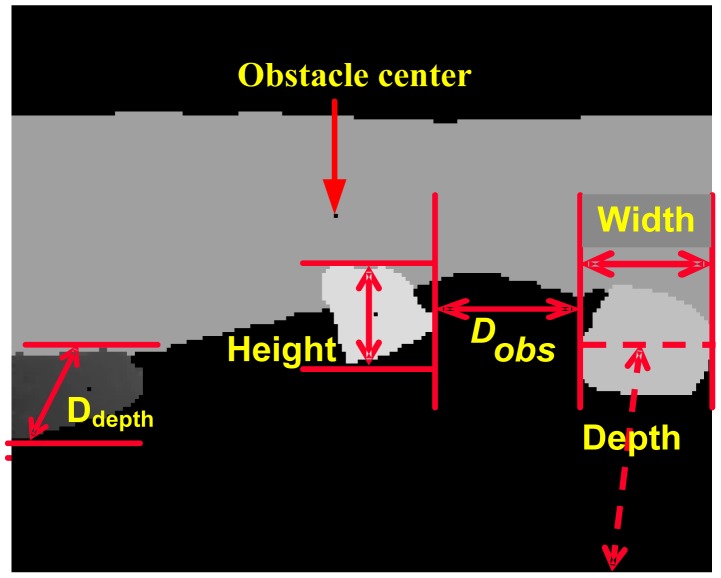
Geometrical features of obstacles.

Each obstacle is characterized by its geometry features. However, natural obstacles may have an irregular shape. Thus, a feature vector {*Width*,*Height*,*R_LH_*,*D_depth_Depth*,*Area*} is selected to describe width, height, ratio between length and height, the maximal depth difference, and the average depth and area of the obstacle, as illustrated in Figure 7 and defined as follows.

*Width:* The maximum horizontal width between the rightmost and the leftmost points in each row within the region:

(11) $$\text{Width}=\max\left(X(i,j_{R})-X(i,j_{L})\right)$$

*Height*: The maximum height difference between the ground and each point within the obstacle is the region's height:

(12) $$\text{Height}=\max(Y(i,j)-H_{g})$$

Ratio between Width and Height:

(13) $$R_{LH}=\text{Width}/\text{Height}$$

*D_depth_*: The maximum difference of depth value of obstacle or object region, which stands for the obstacle's distance range in the direction of depth. As for ditch obstacle, D_depth_ is defined as the range difference between two regions where ground and ditch meeting respectively:

(14) $$D_{\text{depth}}=\text{Max}\left(Z(i_{\text{near}},j_{\text{near}})-Z(i_{\text{far}},j_{\text{far}})\right)$$

*Depth*: the distance between robot and obstacle. The average value of distances of all pixels in the object region stands for the object Depth. In addition, the Depth definition is a little different for a slope obstacle compared with other obstacles. Depth of slope is defined as the average distance of the region where there is a slope and ground effect since slope regions usually have a big range of distance distributions:

(15) $$\text{Depth}=\sum_{\Omega }Z(i,j)/P$$

*Area*: It is clear that the same object would have different amounts of pixels in its image if the object is at different distances to the camera. The relationship between the object area and its depth is generally linear. Consequently, we define the area as a feature related to the true area instead of the ground truth area:

(16) Area=P∗Depth

Similarly, other features about obstacles can also be calculated to describe its location and spatial relationships:

*Distance between obstacles*: For neighboring obstacles, *D_obs_* is defined as the distance between the most right point (*i_R_*,*j_R_*) of the left obstacle and the most left point (*i_L_*,*j_L_*) of the right obstacle without consideration of the height difference:

(17) $$D_{\text{obs}}=\sqrt{{\left(X(i_{L},j_{L})-X(i_{R},j_{R})\right)}^{2}+{\left(Z(i_{L},j_{L})-Z(i_{R},j_{R})\right)}^{2}}$$

*Obstacle center*: Obstacle's location is defined as its gravity center (*i_c_*,*j_c_*) in the intensity image:

(18) $$i_{c}=\frac{1}{M}\overset{d}{\underset{c}{\text{∑}}}\overset{b}{\underset{a}{\text{∑}}}GI(i,j)*i\begin{array}{cc} , & j_{c}=\frac{1}{M}\overset{d}{\underset{c}{\text{∑}}}\overset{b}{\underset{a}{\text{∑}}}GI(i,j)*j \end{array}\begin{array}{cc} , & M= \end{array}\overset{d}{\underset{c}{\text{∑}}}\overset{b}{\underset{a}{\text{∑}}}GI(i,j)$$

#### 3.3.3. Obstacle Recognition Based on Multi-RVM

According to the definition in Section 3.3.1, possible obstacles are classified into four classes. As obstacles like ditches are under the ground plane, this kind of obstacles can be distinguished easily from other three classes of obstacles based on the height difference with the ground plane. As obstacles in the terrain or field environment vary greatly in shape, it is difficult to acquire enough samples for all possible obstacles. Essentially, obstacles recognition subjects to the problem of small sample and nonlinear pattern recognition.

RVM is a machine learning technique that uses Bayesian inference to obtain solutions for classification. This method depends only on a subset of the training data and is able to solve nonlinear pattern recognition problems. RVM has an identical functional form to the support vector machine, but provides probabilistic classification. Compared to SVM, the Bayesian formulation of the RVM avoids the set of free parameters of the SVM (that usually require cross-validation-based post-optimizations), and has less relevant vectors than support vectors given the same prediction performance. Based on these advantages, RVM is used as the obstacle classifier in this work.

**Figure 8.**
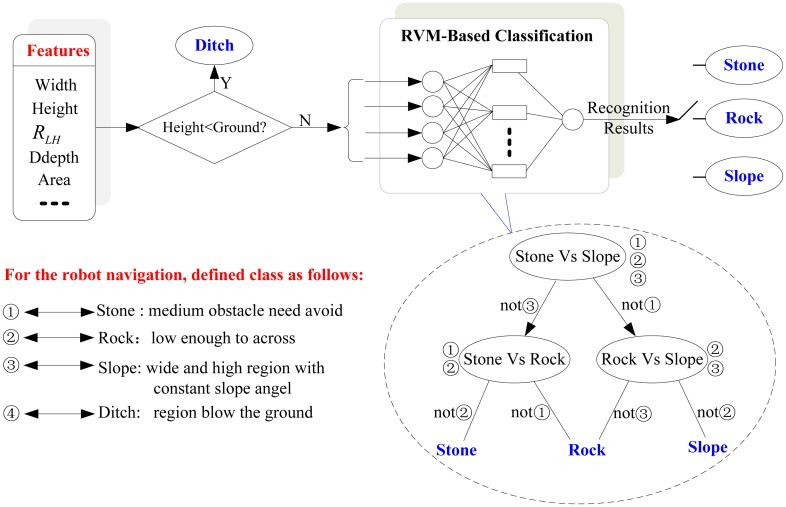
Scheme of obstacles classification based on multi-RVM.

Since we must solve a multi-class problem, the system needs a multi-RVM classifier. We adopt “one-versus-one” method of multi-RVM. In this method, we will construct all the possible two-class classifiers for three kinds of training samples. Every classifier will be trained on two-class training samples of three classes of obstacles. Therefore, six sub-RVM classifiers for two-class classification are needed. Decision is made by a max-wins voting strategy, in which every classifier assigns the instance to one of two classes. Then, the vote for the assigned class is increased by one vote, and finally the class with the most votes determines the instance classification. A detailed scheme is shown in Figure 8.

## 4. Experiments and Data Analysis

**Figure 9.**
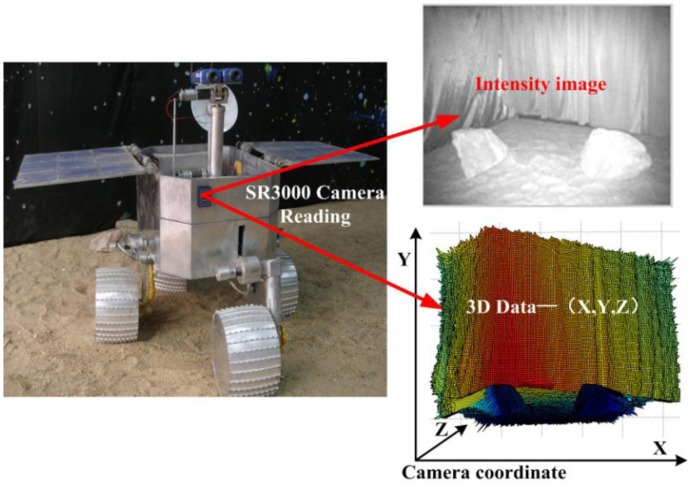
Self-developed mobile robot. The robot is equipped with a SR3000 camera horizontally which simultaneously produces both an intensity image and 3D data with resolution 176 × 144 pixels in real time. The robot can climb obstacles such as steps of 15 cm high, ditches of 10 cm wide, and slopes of 45 degree. It can even walk across obstacles higher than 15 cm with a certain slope angle.

To verify the proposed method, extensive experiments were conducted in different terrain environments based on our self-developed mobile robot shown in Figure 9. In Section 4.1, the obstacle detection results in five different scenes are presented to test the obstacle detecting performance of the proposed method. Then, the feasibility and efficiency of the multi-RVM classifier are evaluated in Section 4.2.

### 4.1. Obstacle Detection and Feature Computation

**Figure 10.**
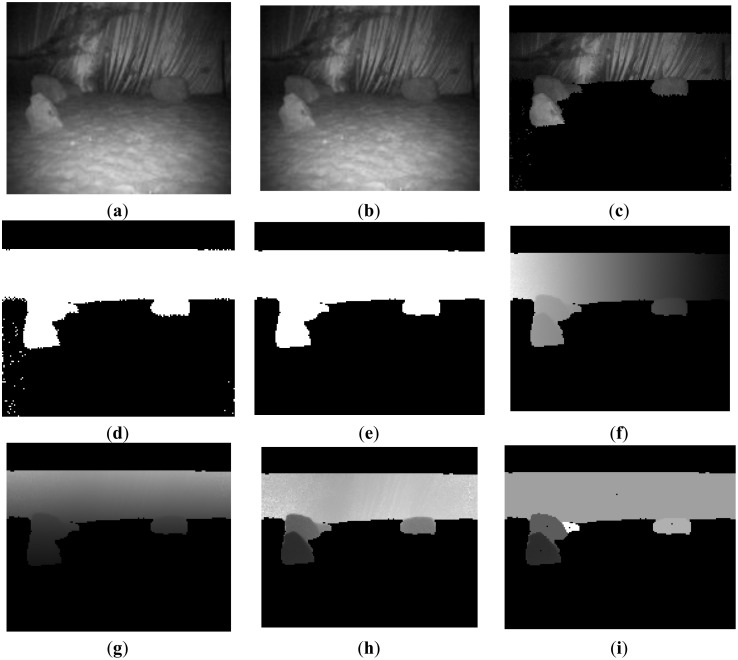
Obstacle detection process and results of the proposed method in Scene 1. (**a**) Original image (**b**) Far Background removed (**c**) Ground and sky region removed; (**d**) Binary image of ROI (**e**) Image erosion and dilation (**f**) X dimension information of scene; (**g**) Y dimension information of scene (**h**) Z dimension information of scene (**i**) Detected obstacles.

**Figure 11.**
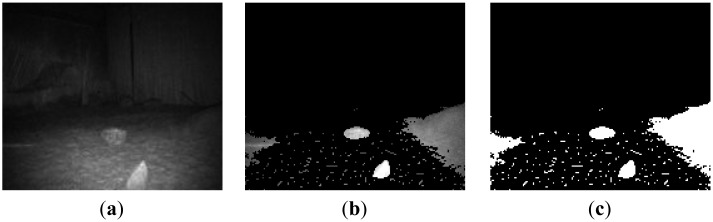

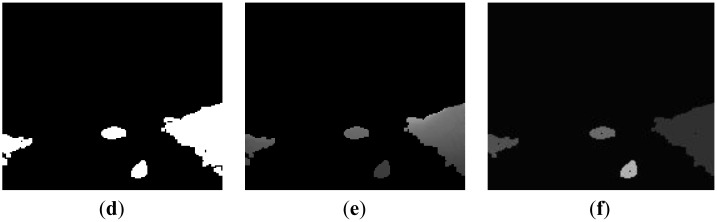
Obstacle detection process and results of proposed method in Scene 2. (**a**) Original image (**b**) Irrelevant region removed (**c**) Binary image of ROI; (**d**) Image erosion and dilation (**e**) Z dimension information of scene (**f**) Detected obstacles.

**Figure 12.**
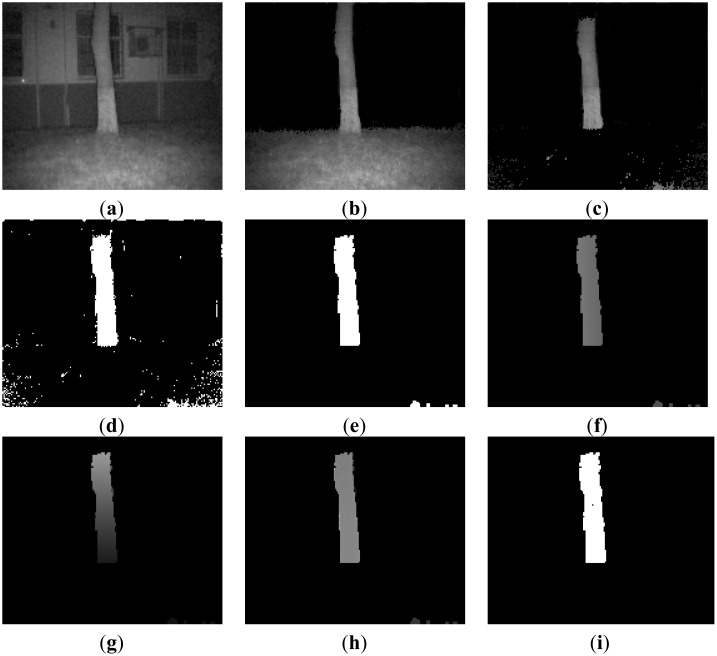
Obstacle detection process and results of proposed method in Scene 3. (**a**) Original image (**b**) Far background removed (**c**) Ground and sky region removed; (**d**) Binary image of ROI (**e**) Image erosion and dilation (**f**) X dimension information of scene; (**g**) Y dimension information of scene (**h**) Z dimension information of scene (**i**) Detected obstacles.

**Figure 13.**
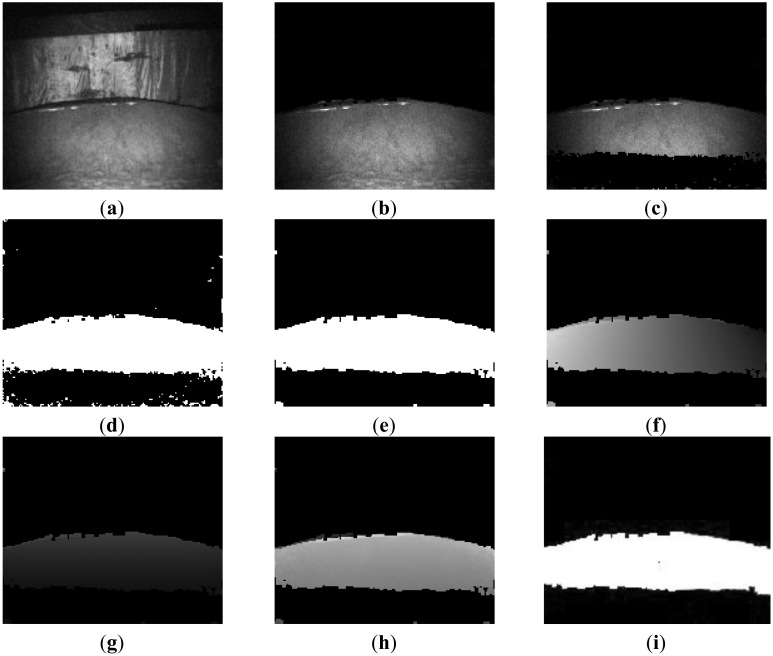
Obstacle detection process and results of proposed method in Scene 4. (**a**) Original image (**b**) Far background removed (**c**) Ground and sky region removed; (**d**) Binary image of ROI (**e**) Image erosion and dilation (**f**) X dimension information of scene; (**g**) Y dimension information of scene (**h**) Z dimension information of scene (**i**) Detected obstacles.

**Figure 14.**
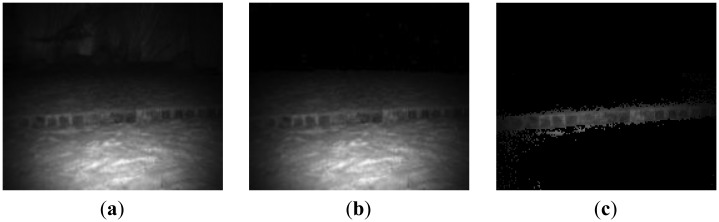

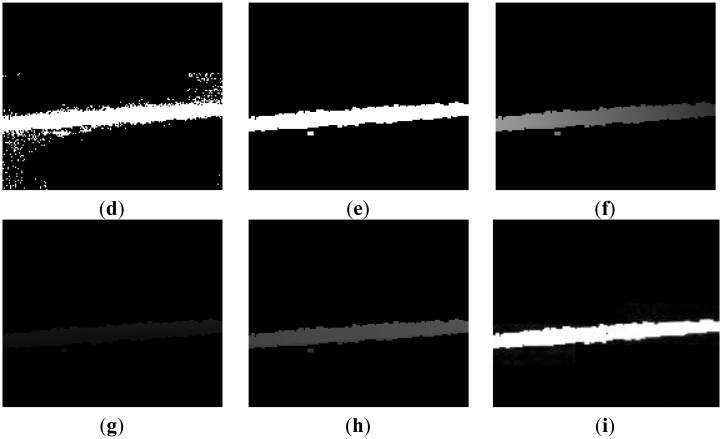
The obstacle detection process and results of proposed method in Scene 5. (**a**) Original image (**b**) Far background removed (**c**) Ground and sky region removed; (**d**) Binary image of ROI (**e**) Image erosion and dilation (**f**) Horizontal information of scene; (**g**) Height information of scene (**h**) Depth information of scene (**i**) Detected obstacles.

Experimental results from five different scenes are presented. The parameters used are *H_S_* = 4*m*, *D_FB_* = 7m, *c*_1_ = 0.15, *c*_2_ = 0.2, *c*_3_ = 0.65. In our results, black areas stand for irrelevant regions, and different obstacles in regions of interest are distinguished using different colors except black. The definition about geometrical parameter (Width, Height, Depth, D_depth_ and Obstacle Center) is presented in Section 3.3.2. The center of detected obstacle is marked as black pixels in Figures 10i, 11f and 12i, 13i and 14i respectively. In addition, detected obstacles in the experiment were used as samples to train the multi-RVM classifier for obstacle recognition. Related parameters for obstacles labeling are H_rc_ = 0.15 m, H_r_ = 0.30 m, W_sl_ = 1.20 m which are set based on the movement performance and characters of the robot. Consequently, the class type of obstacle is labeled according to the obstacle definitions in Section 3.3.1 and related human-labeled data.

#### 4.1.1. Experiment in Scene 1: Stones and Rocks with Occlusion

This environment was a specific scene with a ground with sands, stones and a back wall. The ground was not very flat, and the color difference was not obvious with obstacles. In addition, some obstacles were overlapped in the intensity image.

According to the proposed method, the whole obstacle detecting process is shown in Figure 10. The original intensity image of the scene is shown in Figure 10a. The maximum distance of this scene is smaller than far background threshold (7 m).Therefore no pixels in this scene can be removed in this step of far background elimination. Following this step, the far background region, ground, and sky region respectively were removed, as shown in Figure 10b,c and regions of interest were segmented. The binary image of the region of interest is illustrated in Figure 10d. Consequently, image erosion and dilation were applied to the binary image of the region of interest to filter out small objects or suppress noise. The result is shown in Figure 10e. Finally, based on the 3D geometric information, the detected regions of interest are clustered into separate obstacles as shown in Figure 10f–h. The detected obstacles are shown in Figure 10i.

The result shows that five obstacles were completely detected, even for the very small and overlapped stones. Obstacle feature parameters were also computed in order to test the accuracy of the presented method. The ground truth data were measured manually. The relative error between the measurement value and human labeled data value is shown in Table 2. The length measurement of the 3rd obstacle had a very big relative error with human labeled data, as it was partly occluded by the 2nd obstacle in the horizontal direction.

#### 4.1.2. Experiment in Scene 2: Small Stones and Convex Sand Region

As mentioned in the above section, the ground is often imperfect and not planar in most terrain environments. If the ground height is defined with a narrow range, some ground regions will be detected as obstacles. However, if the ground height is set with a big range, some obstacles will probably not be detected correctly if their height is smaller than the height range. This scene was designed to evaluate the performance of the proposed method in small obstacles detection which height is below 30 cm.

As shown in Figure 11, the presented method gives a satisfactory detection result. Firstly, far background, ground plane and sky region were detected and removed from the scene as shown in Figure 11b. Two small obstacles and two convex regions with little slope angle were detected correctly as shown in Figure 11f. The measurement error was also in acceptable range, as shown in Table 3.

#### 4.1.3. Experiment in Scene 3: Trees and Grass in Natural Environment

This scene is a typical natural outdoor environment. The uneven ground is randomly covered with grass and trees. As shown in Figure 12, firstly the house is separated from the image as its distance is far beyond the Far-Background threshold, and then tree is detected as obstacle after ground detection region clustering.

As shown in Table 4, the height measured by robot looks a bit different from its manual reference value. The reason consisted of two sides, the first is that the sky threshold was just selected as the reference ground truth; the other reason was that the ground is uneven and the detected region became a little shorter after ground deletion and region clustering.

#### 4.1.4. Experiment in Scene 4: Slope

There were three parts in this scene, a wall (Far-background), a ground and a slope made of sands. As the texture and color for ground and slope were same, it was difficult for a robot to distinguish each other depending on 2D images. However, it was very easy to detect slope region based on the proposed method, the detail process is shown in Figure 13. The computed parameters of obstacle and its relative error with human labeled data are shown in Table 5.

#### 4.1.5. Experiment in Scene 5: Ditch in Field Environment

In this scene there was a ditch under the ground. Once again, it was easily detected by the proposed method, the detail steps and results are shown in Figure 14. D_depth_ in Table 6 is the range difference between two regions where ground and ditch are meeting, respectively, which stands for the ditch's width in the depth's direction. Depth is still defined as the average distance of all points in region. As for the human labeled data, we sampled the distance along the ditch evenly, and made an average as a reference value. This definition is coherent with the definition of other obstacles. In addition, the computed parameters of obstacle and its relative error with human labeled data are shown in Table 6.

### 4.2. Experiment of Multi-RVM Obstacle Recognition

#### 4.2.1. Multi-RVM Training

Five hundred scenes with different obstacles were selected as training samples. Based on the proposed obstacle detection method, obstacles and their feature were detected in every scene. As shown in Tables 2, 3, 4, 5 and 6, every sample includes obstacle features and its class type which is denoted by vector {*Width*, *Height*, *R_LH_*, *D_depth_*, *Area*, *Class*}. The number of extracted training set and testing set from 500 scenes is listed in Table 7. In addition, the performances of the presented method and SVM based method were compared and analyzed.

The RBF kernel function was applied for multi-RVM based obstacle classification, as shown in Equation (19):

(19) $$\text{K}(\text{x},{\text{x}}_{\text{i}})=\exp(-\gamma {\left|\text{x}-{\text{x}}_{\text{j}}\right|}^{2})$$

#### 4.2.2. Obstacle Classification Results and Analysis

When the RVM classification method was trained, obstacles were classified and recognized by imputing detected obstacle regions. For test samples with *γ* = 0.1, the classification results are shown in Table 8.

The test set recognition results are shown as in Tables 9 and 10 in which the classifier was trained with different parameters, Table 9 shows the results of the SVM-based classifier with the same training parameters. Table 10 presents the test set recognition result with the RVM-based classifier, where different parameters were used to train the classifier. The experiment results show that the RV number was greatly reduced compared to the SV number. As a result, the processing time of the proposed method was also shorter than that of the SVM-based method. The recognition rate of proposed method was just slightly lower than that of the SVM-based method.

#### 4.2.3. Experiment Result Analysis of Obstacle Detection and Measurement

Experiments have been done to test the accuracy of the proposed method on obstacle classification and geometrical feature measurement. Experimental results in the presented five typical scenes showed that the proposed method can detect obstacles as well as provide valid and dense 3D geometrical measurement with high efficiency and robustness, which is critical for intelligent robot navigation. In addition, experimental results are analyzed in detail as follows:

(1) This method has a very high robustness in complex and unstructured environments. Firstly, in our tested environments, the obstacle classification accuracy of the proposed method maintains a high level. As shown in Table 10, the recognition accuracy of obstacles reaches 93%. Secondly, the relative error illustrated in Tables 2, 3, 4, 5 and 6 is usually lower than 5% except for some special overlapped or very irregular cases in Tables 2 and 3. On the other hand, since the ground truth is provided by human labeling, it has some inherent measurement error, especially for irregular or overlapped obstacles in complex environments. As a result, the real relative accuracy of the presented method may be higher or lower than the data in above experiments, which reflects a trend of accuracy.
(2) The accuracy of the proposed method can be improved in the future. As the ToF camera has low resolution either in the 2D intensity image output or 3D depth image, compared with the enhancement on range accuracy [24,28–34], it is also very necessary to improve the accuracy on obstacle segmentation. As for our proposed method, the accuracy of obstacle geometrical parameters, such as Width, Height, D_obs_, are greatly affected by the object segmentation accuracy. In the future, we will consider the fusion of ToF camera with a 2D high-resolution camera. This will produce high quality color image as well as corresponding depth image, which will improve the segmentation accuracy of obstacles further.
(3) The proposed method is highly efficient and easy to implement. It can finish the process of obstacle recognition and 3D measurement within the time of a single frame of the ToF camera data without any prior knowledge. In addition, the method is easy to implement as the whole process consists of only simple operations.

## 5. Comparison and Discussion

Compared with existing laser or stereo-vision based obstacle detection methods, our method presents a new ToF-camera based obstacle recognition and measurement solution inspired by human 3D vision perception. As no public databases with the abovementioned different sensors in same scenes are available, it is difficult to conduct a comparison test using the same environment. Therefore, in this section, we presents a performance and advantage analysis based on published technical data of the existing methods.

Laser sensor based methods are efficient in obstacle detection because its readings focus on specified height and direction. This process is fast and accurate. However, laser sensor reading on the whole 3D space is sparse and time-consuming, which greatly limits the applications of this method to obstacle classification and 3D measurement. As a result, this paper does not present a comparison between the proposed method and the laser-based methods.

Since the stereo-vision system can provide both an intensity image and the depth information, it has been widely used in obstacle recognition and measurement. The technical characteristic comparison between the proposed method and stereo-vision based methods is presented as follows along with the results listed in Table 11:

(1) The presented method can recognize more obstacle classes with higher efficiency than stereo-vision based methods. Based on one single frame, as shown in Table 11, the proposed method can recognize four types of obstacles while the methods reported in [23,28,29] can only one or two obstacles. In addition, the 3D information computing process is complex and the quality of the 3D information varies in different scenes. Consequently, stereo-vision based methods are usually used for obstacle detection and simple classification. For example, the method in [28] can only detect rocks, and the methods in [23,29] can make a distinction between large rocks and small rocks. To improve the classification performance, some methods introduced more stereo frames. In [19], obstacles are classified into “Obstacles”, “Negative obstacle” and “irrelevant” from three stereo image frames. In [37], environment is semantically labeled as Level, Ground, Vertical, Stairs based on 3D map model of the scene built by registered stereo data sequence. As a result, the proposed method is more advantageous in accurate obstacle subdivision compared with single frame based stereo methods. In addition, our method is computing and memory efficient compared with multiple frames based stereo methods.
(2) The proposed method has a higher accuracy in obstacle identification based on a single frame. As shown in Table 11, the proposed method reaches 93% in average accuracy for four obstacle classes, while the method in [28] reached 89% for one obstacle class. A more advanced method [29] reached 90% for two obstacle classes.
(3) (3) The proposed method has obvious advantages in 3D geometrical measurement of obstacles. As illustrated in Section 4, we make use of 3D information and intensity image for detecting obstacles, and provide high resolution 3D information of detected obstacles. Although stereo vision based methods also allow efficient detection of obstacles, the limited depth resolution and depth accuracy decrease as the distance between camera and obstacle increase. This problem imposes a strong restriction for geometrical feature measurement of obstacles. The method [37] provides 3D information of detected obstacles depending on a 3D map reconstructed from stereo image sequence of the environment rather than single stereo frame.

## 6. Conclusions

This paper presents a method for real-time obstacle detection and recognition in unstructured environments based on the use of a ToF camera. The proposed method defines regions of interest to guide navigation, simulating the perception mechanism in human navigation. Based on the 3D data acquired by the SR 3000 ToF camera, we first perform a detection of irrelevant regions based on the geometrical characteristics of certain objects irrelevant to navigation, such as far background, sky and the ground which do not affect the movement of a robot. Then, the obstacles as other regions of interest are detected ignoring the irrelevant regions from the input scene. After this step, the 3D geometrical parameters and other features of every detected obstacle are computed for classification. Finally, considering possible irregular obstacle shapes and the movement performance of the robot, a multi-RVM classifier is designed to classify obstacles into four classes. Experimental results on obstacle detection in different scenes indicate that our method can detect obstacles robustly, and it even works well for small obstacles with height below 30 cm and situations with a certain occlusion. In addition, the relative error of obstacle features with human labeled measurement was found to be in a similar small error range of our autonomous navigation, and the proposed multi-RVM classifier produces a satisfactory recognition rate with a very small RV number.

This work provides a robust, simple and high efficiency solution for practical robotic navigation problems involving 3D obstacles in unknown, unstructured environments. However, the resolution, the quality of the intensity image, and the range measurement accuracy of the ToF camera (SR3000) are currently low, which directly limits the accuracy in geometrical measurements and classification of obstacles. Besides improvements on range accuracy, we will improve the accuracy of obstacle measurement and classification further by fusing a 2D color camera with the ToF camera to produce both high-quality image and the corresponding depth information.

## Figures and Tables

**Table 1.** The definition of region of interest and irrelevant region.

**Region Type**	**Definition**	**Specification**	**Operation**
Sky	Region is higher than robot can reach	Irrelevant region for robot navigation	Remove it from the scene

Far background	Region is far from the robot in the heading direction, and it has little effect on local navigation behavior		

Ground	Flat region where robot can walk safely

Obstacles	Regions where robot has difficulty in walking through, such as ditch, rock, tree, wall, inclines, *etc*.	Regions of interest (ROI) for robot navigation	Detection, measurement and classification

**Table 2.** Detected obstacles and its 3D parameters by proposed method in scene 1.

**Obstacle**	**Class Type**	**Width×Height×Depth**		

		**Measurement by Robot (cm)**	**Human Labeled Data (cm)**	**Relative Error (%)**
1-Left-back	Stone	40 × 41 × 230	39 × 42 × 233	2.6 × 2.3 × 1.3
2-Left-middle	Stone	39 × 41 × 273	40 × 42 × 271	2.5 × 2.3 × 0.7
3-Left-last	Rock	18 × 13 × 297	27 × 14 × 300	33.3 × 7.1 × 1.0
4-Right	Stone	46 × 25 × 376	48 × 26 × 376	4.1 × 3.8 × 0.0

**Table 3.** Detected obstacles and its 3D parameters by proposed method in Scene 2.

**Obstacle**	**Class Type**	**Width×Height×Depth**		

		**Measurement by Robot (cm)**	**Human Labeled Data (cm)**	**Relative Error (%)**
1-Left	Rock	52 × 19 × 350	60 × 16 × 359	13.3 × 18.7 × 2.5
2-Left-center	Rock	29 × 18 × 253	34 × 20 × 255	17.6 × 10 × 0.8
3-Right-center	Rock	19 × 25 × 215	23 × 27 × 211	17.3 × 7.4 × 1.9
4-Right	Stone	105 × 57 × 376	110 × 53 × 366	4.6 × 7.6 × 2.2

**Table 4.** Detected obstacles and its 3D parameters by proposed method in Scene 3.

**Obstacle**	**Class Type**	**Width×Height×Depth**		

		**Measurement by Robot (cm)**	**Human Labeled Data (cm)**	**Relative Error (%)**
1	Stone	31 × 379 × 435	33 × 400 × 433	6.1 × 5.3 × 0.5

**Table 5.** Detected obstacles and its 3D parameters by proposed method in Scene 4.

**Obstacle**	**Class Type**	**Width×Height×Depth**		

		**Measurement by Robot (cm)**	**Human Labeled Data (cm)**	**Relative Error (%)**
1	Slope	418 × 132 × 574	430 × 129×578	2.8 × 2.3 × 0.7

**Table 6.** Detected obstacles and its 3D parameters by proposed method in Scene 5.

**Obstacle**	**Class Type**	**Width×D_depth_×Depth**		

		**Measure by Robot (cm)**	**Human Labeled Data (cm)**	**Relative Error (%)**
1	Ditch	352 × 35 × 221	360 × 37 × 225	2.2 × 5.4 × 1.8

**Table 7.** Numbers of training set and testing set for different obstacle class.

**Obstacle Class**	**Number of Training Set**	**Number of Testing Set**
Rock	238	163
stone	282	201
Slope	167	114
Total	687	478

**Table 8.** Recognition result of testing sample set using multi-RVM with *γ* = 0.1.

**Obstacle Class**	**Number of Sample Set**	**Accuracy**
Rock	163	92.7%
stone	201	90.4%
Slope	114	95.5%
Total	478	92.86%

**Table 9.** Recognition accuracy and SV number of multi-SVM with different σ.

**Kernel Parameter** σ	**C**	**Recognition accuracy**	**SV Number**
0.1	1000	93.62%	322
1	65	94.07%	338
1.74	40	95.85%	353
1.74	1000	95.56%	335

**Table 10.** Recognition accuracy and RV number of multi-RVM with different σ.

**Kernel Parameterσ**	**Recognition Accuracy**	**RV Number**
0.1	92.86%	45
0.7	93.40%	56
1	93.67%	61
1.74	93.24%	50

**Table 11.** Technical comparisons between the proposed method and stereo-vision based methods. (“—” indicates corresponding method does not provide this item).

**Methods Compared Items**	**Proposed Method**	**V. Gor [23]**	**R. Castano [28]**	**P. Santana [29]**	**T. Dang [19]**	**R. Bogdan [37]**
Sensors	ToF camera	Stereo camera	Stereo camera	Stereo camera	Stereo camera	Stereo camera
Recognized obstacle types	Four types: Stone/Rock/Slope/Ditch	Two types: Small rocks/large rocks	One type: Rock	Two types: large obstacles/small obstacles	Two types: obstacle/negative obstacle” irrelevant	Tree types: Level(Slope)/Vertical/Stairs
Frames needed for obstacle recognition	Single ToF-camera Frame	Single Stereo Frame	Single Stereo Frame	Single Stereo Frame	Three consecutive stereo frames	Multiple stereo frames to build 3D model of the scene
Computing efficiency	real time	Near real time	Near real time	Near real time	Not real time	Not real time
Classification accuracy	About 93%	Low	About 89%	90% in average	—	—
3D information of Object	Accurate 3D information available	—	—	—	—	3D information available
